# Visible light-promoted transition metal-free direct C3-carbamoylation of 2*H*-Indazoles

**DOI:** 10.3389/fchem.2022.1087834

**Published:** 2022-11-29

**Authors:** Chunhua Ma, Linchun Shang, Hanying Zhao, Xing He, Qiyan Lv, Dandan Zhang, Yuqin Jiang

**Affiliations:** ^1^ Collaborative Innovation Centre of Henan Province for Green Manufacturing of Fine Chemicals, Key Laboratory of Green Chemical Media and Reactions, Ministry of Education, Henan Engineering Research Centre of Chiral Hydroxyl Pharmaceutical, Henan Engineering Laboratory of Chemical Pharmaceutical and Biomedical Materials, School of Chemistry and Chemical Engineering, Henan Normal University, Xinxiang, China; ^2^ National Engineering Research Center of Low-Carbon Processing and Utilization of Forest Biomass, Nanjing Forestry University, Nanjing, China; ^3^ Green Catalysis Center, College of Chemistry, Zhengzhou University, Zhengzhou, China

**Keywords:** photocatalysis, 2H-indazole, carbamoylation, green oxidant, antitumor

## Abstract

We reported a general transition metal-free transformation to access C3-carbamoylated 2*H*-indazoles via visible light-induced oxidative decarboxylation coupling, in the presence of oxamic acids as the coupling sources, 4CzIPN as the photocatalyst, and Cs_2_CO_3_ as the base. The great application potential of this mild condition is highlighted by the late-stage modification of drugs, N-terminal modification of peptides, and the good antitumor activity of the novel desired product.

## Introduction

**FIGURE 1:**
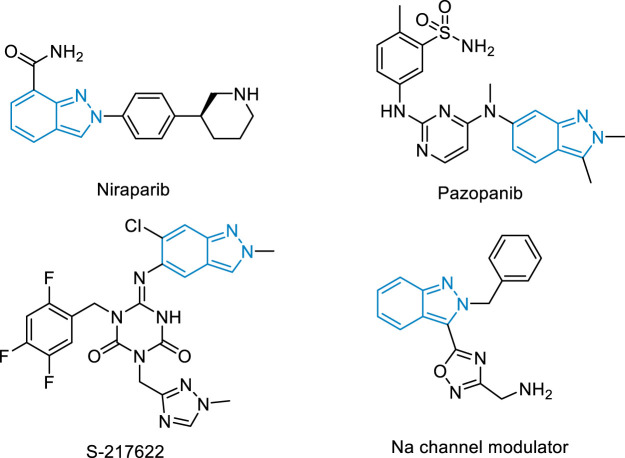
The drugs and bioactive molecules with 2*H*-indazoles.

Nitrogen heterocycles are the essential structural elements widely ubiquitous in pharmaceutical chemistry, (Vitaku et al., 2014; Bhutani et al., 2021; Ma et al., 2021a), organic chemistry, (Chen et al., 2021; Darroudi et al., 2021; Jiang et al., 2021; Meng et al., 2021; Qu et al., 2021; Wang and Wang, 2021; Chen and Xuan, 2022; Gao et al., 2022; Wu et al., 2022; Zhang et al., 2022), and material chemistry (Huang and Yu, 2021). Among these, 2*H*-indazole is one of the most important heterocycles, existing in various drugs and bioactive molecules (Figure 1). The drug Niraparib with this scaffold is approved to treat various tumors including advanced epithelial ovarian carcinoma and primary peritoneal carcinoma. (Jones et al., 2009). The derivative Pazopanib has become the first-line anti-advanced renal cell carcinoma *via* inhibiting the activity of vascular endothelial growth factor receptor VEGFR. (Harris et al., 2008). The 3C-like protease inhibitor S-217622 has entered into clinical trials and exhibits antiviral activity against the coronavirus disease 2019 (COVID-19). (Unoh et al., 2022). Therefore, direct and site-selective incorporation of diverse functional groups into 2*H*-indazole is of broad interest in organic synthesis and the pharmaceutical industry.

**Scheme 1:**
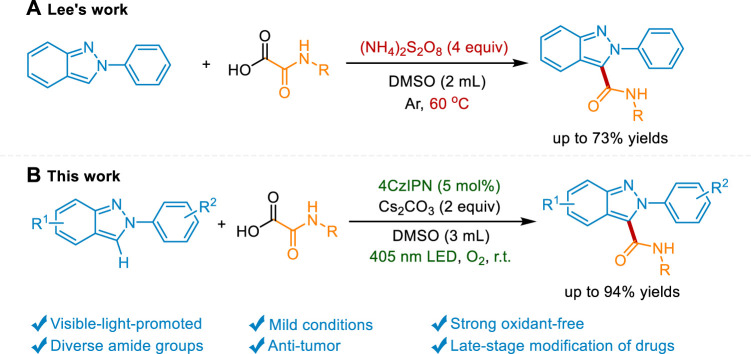
Synthetic approaches to C3-carbamoylation of 2*H*-indazoles.

Recent decades have witnessed the impressive achievement of direct C-H functionalization of 2*H*-indazoles *via* radical reactions. (Ghosh et al., 2020; Wang et al., 2022a; Ghosh et al., 2022). The C3-phosphonylation, (Singsardar et al., 2018), oxyalkylation, (Singsardar et al., 2019), trifluoromethylation, (Murugan et al., 2019; Wei et al., 2021), amination, (Neogi et al., 2020; Sun et al., 2021a), alkoxylation, (Sun et al., 2021b), arylation, (Aganda et al., 2019; Vidyacharan et al., 2019; Saritha et al., 2021), alkylation, (Liu et al., 2020; Ma et al., 2021b; Ma et al., 2022a), sulfonylation, (Kim et al., 2020; Mahanty et al., 2020), and selenylation (Lin et al., 2022) of 2*H*-indazole were reported. However, the development of sustainable strategies to introduce other pharmacophores into 2*H*-indazole is still highly desirable. Amide groups represent a fundamental class of functional groups widely spread in most drugs, bioactive compounds, and peptides. Compared with the traditional condensation method, the C-H carbamoylation protocol provides the desired product without prefunctionalization of the 2*H*-indazole and wasteful coupling reagents. Nevertheless, the direct carbamoylation of 2*H*-indazole is rarely reported. Only recently, Lee’s group reports an elegant carbamoylation reaction of 2*H*-indazole using oxamic acid as a carbamoylating source under an elevated temperature in the presence of the strong oxidant (NH_4_)S_2_O_8_. (Bhat and Lee, 2021). However, the heating process which is essential for the radical generation results in the consumption of fossil fuels and the potential safety hazard. Meanwhile, a great quantity of strong oxidant might be detrimental to the sensitive functional groups. Photocatalysis has emerged as a strong strategy to the functionalization of the nitrogen heterocycles. (Liu et al., 2017; Bagdi et al., 2020; Yuan et al., 2020; He et al., 2021; Qi et al., 2021; Yi and He, 2021; Ma et al., 2022b; Wang et al., 2022b; Ma et al., 2022c; Ma et al., 2022d; Ma et al., 2022e; Shi et al., 2022; Xiang et al., 2022; Yan et al., 2022; Yang et al., 2022; Zhu et al., 2022). The mild reaction condition and the visible light-induced neutral redox cycle may solve the above problems. Herein, we reported a visible light-mediated strong oxidant-free protocol to access the carbamoylated 2*H*-indazoles under mild conditions and the late-stage modification of drugs and peptides (Scheme 1).

## Results and discussion

We chose 2-phenyl-2*H*-indazole (1a) and 2-(hexylamino)-2-oxoacetic acid (2a) as model substrates to investigate the decarboxylative C (sp^2^)-C (sp^2^) coupling reaction under 405 nm purple LED irradiation at room temperature. Consistent with the expected, when 4CzIPN was used as the photocatalyst and Cs_2_CO_3_ as the base, 1a and 2a could be converted into the carbamoylated 2*H*-indazole 3a in 56% yield under O_2_ atmosphere (Table 1, entry 1). Other transition metal-free photocatalysts including Rhodamine B, Rhodamine 6G, Fluorescein, Na_2_-Eosin Y, and Rose bengal were catalytically inactive, with no product detected (Table 1, entries 2–6). Then, a systematic survey of bases were conducted. The results indicated that replacing Cs_2_CO_3_ with other inorganic bases (Na_2_CO_3_, K_2_CO_3_, LiOH, KOH, CsOH) or organic bases (Et_3_N, DIPEA, TMEDA, DABCO) decreases the formation of the desired product (Table 1, entries 7–15). A range of solvents, such as, DCM, MeCN, DMF, DMAC, NMP, THF, DMC, EG, and H_2_O were screened (Table 1, entries 16–24). We found that DMSO is superior in this process. Increasing the amount of 2a to 2.5 equiv. improved the yield to 74% (Table 1, entry 25). Because the insoluble residue existed in the reaction system, the volume of DMSO was increased to 3 ml, along with the generation of products in 91% yield (Table 1, entry 26). In the absence of visible light or photocatalyst, no product was detected, which confirms the photochemical nature of this method (Table 1, entries 27–28). The reaction was completely inhibited in the absence of Cs_2_CO_3_, indicating the essential role of the base in the transformation (Table 1, entry 29). Taken together, the optimal reaction conditions were established as follows: 1a (0.2 mmol), 2a (2.5 equiv), 4CzIPN (5 mol%) as a catalyst, Cs_2_CO_3_ (2 equiv) as a base, DMSO (3 ml) as a solvent, at 35°C under O_2_ atmosphere and the irradiation of purple LED (*λ*_max_ = 405 nm) for 12 h.

**TABLE 1:** Optimization of reaction conditions^a^.

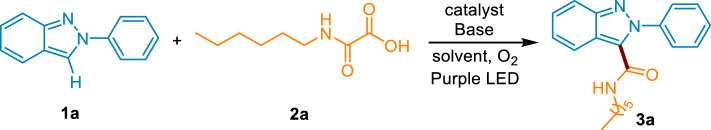				
Entry	Photocatalyst (5 mol%)	Base (2 equiv)	Solvent	Yield (%)
1	4CzIPN	Cs_2_CO_3_	DMSO	56
2	Rhodamine B	Cs_2_CO_3_	DMSO	0
3	Rhodamine 6G	Cs_2_CO_3_	DMSO	0
4	Fluorescein	Cs_2_CO_3_	DMSO	0
5	Na_2_-Eosin Y	Cs_2_CO_3_	DMSO	0
6	Rose bengal	Cs_2_CO_3_	DMSO	0
7	4CzIPN	Na_2_CO_3_	DMSO	23
8	4CzIPN	K_2_CO_3_	DMSO	17
9	4CzIPN	LiOH	DMSO	7
10	4CzIPN	KOH	DMSO	7
11	4CzIPN	CsOH	DMSO	32
12	4CzIPN	Et_3_N	DMSO	10
13	4CzIPN	DIPEA	DMSO	5
14	4CzIPN	TMEDA	DMSO	5
15	4CzIPN	DABCO	DMSO	6
16	4CzIPN	Cs_2_CO_3_	DCM	22
17	4CzIPN	Cs_2_CO_3_	MeCN	0
18	4CzIPN	Cs_2_CO_3_	DMF	3
19	4CzIPN	Cs_2_CO_3_	DMAC	13
20	4CzIPN	Cs_2_CO_3_	NMP	32
21	4CzIPN	Cs_2_CO_3_	THF	0
22	4CzIPN	Cs_2_CO_3_	DMC	0
23	4CzIPN	Cs_2_CO_3_	EG	0
24	4CzIPN	Cs_2_CO_3_	H_2_O	0
25^b^	4CzIPN	Cs_2_CO_3_	DMSO	74
26^c^	4CzIPN	Cs_2_CO_3_	DMSO	91
27^d^	4CzIPN	Cs_2_CO_3_	DMSO	N. R
28^c^	--	Cs_2_CO_3_	DMSO	N. R
29^c^	4CzIPN	--	DMSO	N. R

^a^ Reaction conditions: 1a (0.2 mmol), 2a (2 equiv), catalyst (5 mol%), base (2 equiv), solvent (2 ml), rt, LED, 12 h under O_2_ atmosphere. Isolated yields. N. R. = no reaction.

^b^ 2a (2.5 equiv).

^c^ 2a (2.5 equiv), DMSO (3 ml).

^d^ Without light.

**Scheme 2:**
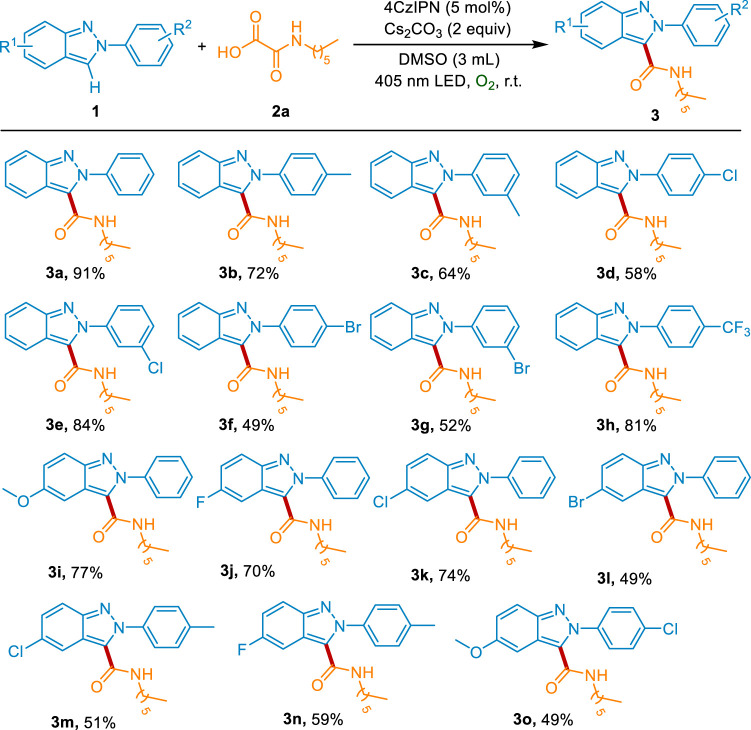
The scope of 2-aryl-2*H*-indazoles.

With the optimal conditions for the construction of carbamoylated 2*H*-indazoles in hand, we further explored the scope and generality of this reaction. Firstly, the scope of aryl-2*H*-indazoles was examined. As shown in Scheme 2, the substitutions on the phenyl group exhibited good tolerance. The electron-donating groups (*p*-Me and *m*-Me) could give the desired products 3b-3c in 72% and 64% yields, respectively. The derivatives with electron-withdrawing groups (*p*-Cl, *m*-Cl, *p*-Br, *m*-Br, and *p*-CF_3_) were also effective substrates for this transformation, affording the corresponding products 3d-3h in moderate to good yields. Moreover, both the electron-donating substitution (5-OMe) and the electron-withdrawing groups (5-F, 5-Cl, and 5-Br) on the heteronucleus were well tolerant to the standard conditions (3i-3l). The 2*H*-indazoles with disubstitution were also evaluated to react with 2a under the optimal condition, delivering the corresponding products 3m-3o in 49–59% yields.

**Scheme 3:**
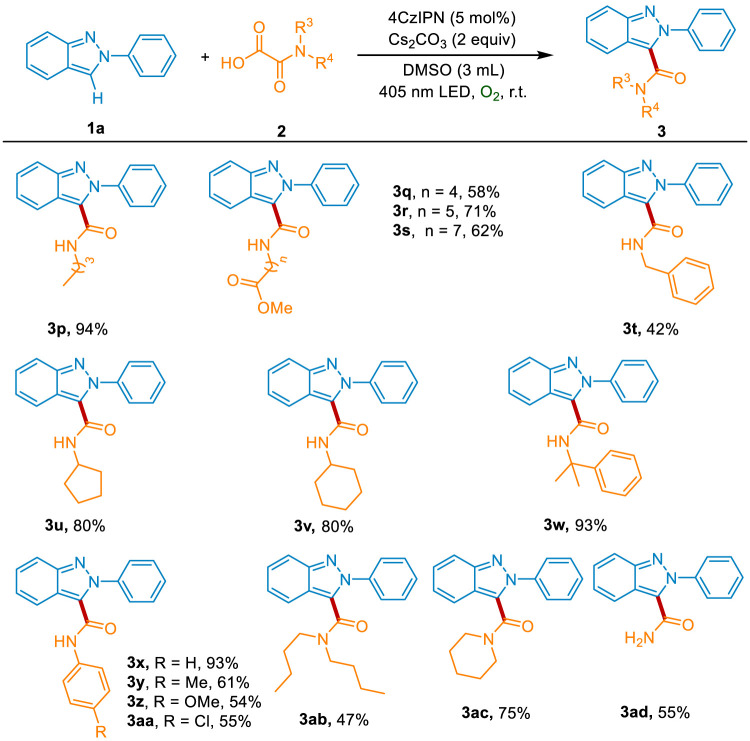
The scope of oxamic acids.

Subsequently, we investigated the reactivity profile of a variety of oxamic acids 2. As depicted in Scheme 3, oxamic acids with different length alkyl chains reacted well with 1a, affording the desired products 3p-3s in 58%–94% yields. The benzyl group was also compatible with the method, giving the product 3t in 42% yield. Both the secondary carbon (cyclopentyl and cyclohexyl group) and tertiary carbon (2-phenylpropyl group) substituted oxamic acids were successful in providing the corresponding products 3u-3w in 80%–93% yields. Meanwhile, the substrates containing primary aromatic amines reacted well with 1a and produced the desired products 3x-3aa in 54%–93% yields. The oxamic acids bearing secondary amine also showed good reactiveness and could be smoothly converted into the carbamoylated products 3ab and 3ac. Moreover, the oxamic acid without N-substitution was tolerated to generate the desired product 3ad in 55% yield.

**Scheme 4:**
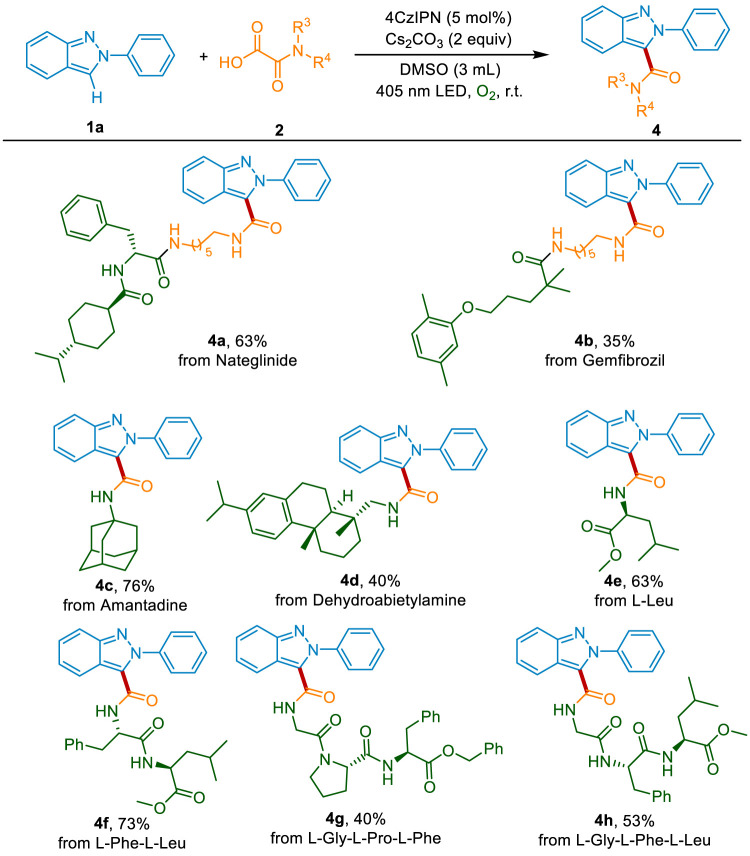
The modification of drugs, natural products, and peptides.

To evaluate the synthetic utility of this decarboxylative carbamoylation transformation in the pharmaceutical industry, the late-stage modification of drugs and natural products was conducted. Delightfully, the non-sulfonylureas antibiabetic drug Nateglinide, the lipid regulator Gemfibrozil, and the antiviral drug amantadine could be successfully connected with 2*H*-indazole, affording the desired products 4a-4c in 35%–76% yield (Scheme 4). The natural product dehydroabietylamine was also suitable and gave the products 4d in 40% yield. The N-terminal modificated of peptides play an important role in drug development and biochemical research. Inspired by the good functional group tolerance of this sustainable system, we then applied the photocatalytic method in the modification of natural amino acids and peptides. As shown in Scheme 4, the important amino acid in humans, l-leucine, could be converted into the corresponding products 4e in 63% yield. What’s more, both the dipeptide (l-phenylalanine-l-leucine) and the tripeptides (l-glycine-l-proline-l-phenylalanine and l-glycine-l-phenylalanine-l-leucine) reacted well with 2*H*-indazole, delivering the coupling products 4f-4h in 40%–73% yields. The above results indicate that this method could be used in the development of peptidomimetic drugs and probe molecules.

**Scheme 5:**
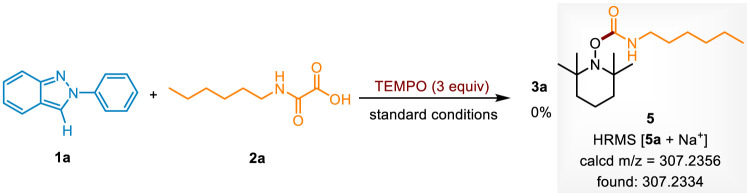
The radical scavenge experiment.

To investigate the mechanism of this carbamoylation reaction, a radical scavenge experiment was conducted (Scheme 5). When 2,2,6,6-tetramethylpiperidinyl-1-oxyl (TEMPO) was added to the standard conditions, the reaction completely shuttled down. Moreover, the carbamoyl radical trapped adduct 5 was detected by HRMS. It indicates that this photocatalytic transformation occurred *via* a radical pathway. Next, it was found that the yields of 3a were decreased to 9% and 23% under N_2_ atmosphere or air atmosphere, revealing that O_2_ is important in the photocatalytic system.

**Scheme 6:**
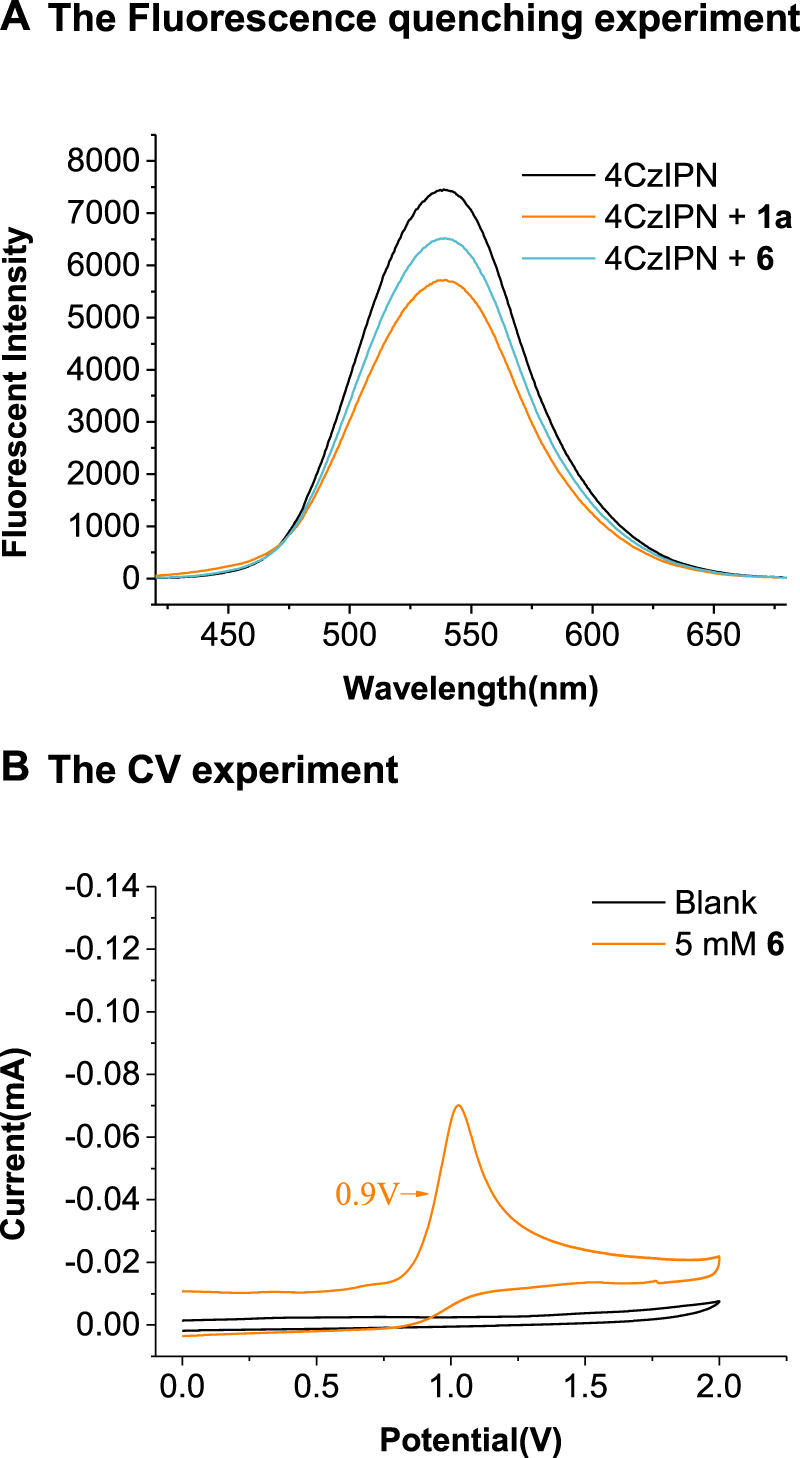
The Stern–Volmer luminescence-quenching experiments and CV experiment.

We performed the Stern–Volmer luminescence-quenching experiments by mixing the photocatalyst 4CzIPN with different concentrations of 2*H*-indazole 1a, 2-(hexylamino)-2-oxoacetic acid 2a, or the Cs salt of 2a (6). As depicted in Scheme 6A, the fluorescence of photoredox catalyst 4CzIPN was quenched by the addition of 1a and 6, and the linear relationships were observed between I_0_/I and the concentration of 1a and 6 (see the Supplementary Figure S2). The oxidative potential of 6 was *E*_1/2_^ox^ = +0.9 V vs*.* SCE (Scheme 6B), indicating that the excited state 4CzIPN (*E*_1/2_(P*/P^−^) = +1.35 V vs*.* SCE) (Shang et al., 2019) could be reductively quenched by 6 rather than 1a (*E*_1/2_^ox^ = +1.4 V vs*.* SCE) (Ma et al., 2021b).

**Scheme 7:**
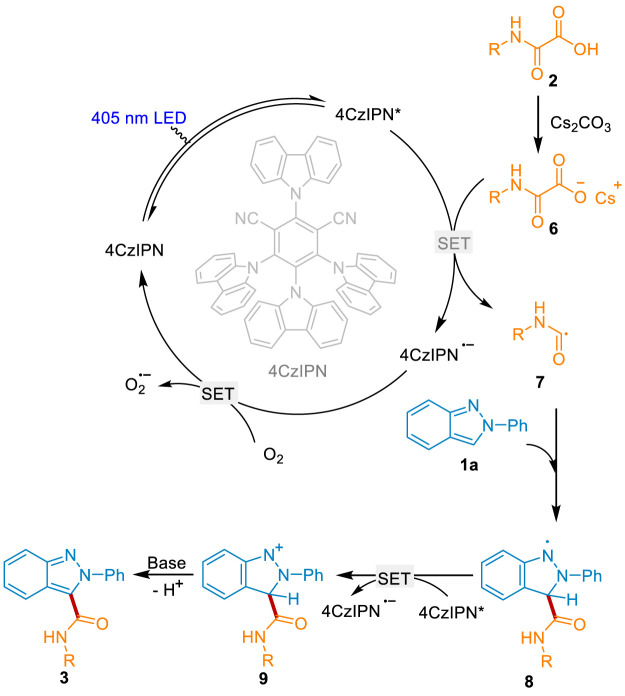
The plausible reaction mechanism.

A plausible mechanism for this sustainable reaction was proposed according to the above experimental results and the previous reports (Scheme 7). Initially, 4CzIPN was activated into the excited state 4CzIPN* under visible light irradiation. The oxamic acid 2 was *in situ* converted into the Cs salt 6 in the presence of the base Cs_2_CO_3_. 6 underwent the oxidization of 4CzIPN* *via* single electron transfer (SET) and fragmentation to generate the key carbamoyl radical 7, along with the production of the radical anion 4CzIPN^•-^. 4CzIPN^•-^ was oxidated by O_2_ to regenerate the ground state 4CzIPN and close the photoredox cycle. On the other hand, radical 7 attacked the C3-position of 1a to deliver intermediate 8. It underwent the 4CzIPN* mediated oxidation and base mediated dehydrogenate to afford the desired product 3.

**Scheme 8:**
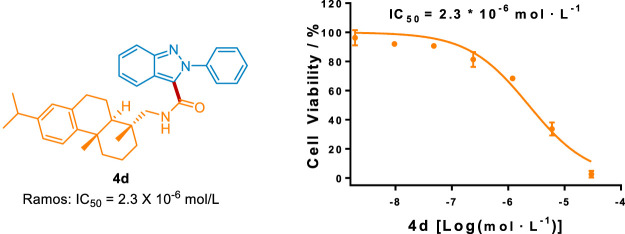
The antitumor activity of 4d against Ramos cell.

To highlight this greener protocol in the pharmaceutical industry, we evaluated the *in vitro* antitumor activity of these carbamoylated 2*H*-indazole derivatives. As depicted in Scheme 8, compound 4d possessed better antitumor activity against Ramos cell than that of the FDA-approved drug 5-fluorouracil (5-FU, IC_50_ = 36.0 × 10^−6^ mol/L), suggesting that this method could provide novel chemical entries for anti-human B cell lymphoma treatment.

## Conclusion

In summary, we have developed a visible-light-promoted, transition metal-free, strong oxidant-free method to achieve the direct decarboxylation/carbamylation of 2-aryl-2*H*-indazoles. This mild and general protocol is tolerant of sensitive functional groups and sterically hindered groups. It is highlighted by the successful application in the late-stage modification of drugs, natural products, amino acids, and peptides. Moreover, the good antitumor activity of compound 4d indicates that this strategy could be used in antitumor drug development. Further activity studies and structural modification are ongoing in our laboratory.

## Data Availability

The original contributions presented in the study are included in the article/Supplementary Material, further inquiries can be directed to the corresponding authors.
