# Supplementary material for: Visible light-promoted transition metal-free direct C3-carbamoylation of 2H-Indazoles
Source: Front Chem. 2022 Nov 29;10:1087834. doi: 10.3389/fchem.2022.1087834 (PMC9745185; doi:10.3389/fchem.2022.1087834)
Supplement: Supplementary file 1 [file DataSheet1.docx]

## Supporting Information

**Visible Light-Promoted Transition Metal-Free Direct C3** **Carbamoylation of 2*H*-Indazoles**

**Chunhua Ma,^1^ Linchun Shang,^1^ Hanying Zhao,^1^ Xing He,^1^ Qiyan Lv,^2,3^ Dandan Zhang,^1^* and Yuqin Jiang^1^***

^1^Collaborative Innovation Centre of Henan Province for Green Manufacturing of Fine Chemicals, Key Laboratory of Green Chemical Media and Reactions, Ministry of Education, Henan Engineering Research Centre of Chiral Hydroxyl Pharmaceutical, Henan Engineering Laboratory of Chemical Pharmaceutical and Biomedical Materials, School of Chemistry and Chemical Engineering, Henan Normal University, Xinxiang 453007, China.

^2^National Engineering Research Center of Low-Carbon Processing and Utilization of Forest Biomass, Nanjing Forestry University, Nanjing 210037, China

^3^Green Catalysis Center, College of Chemistry, Zhengzhou University, Kexue Road No. 100, Zhengzhou 450001, China.

**Table of Contents**

[Supporting Information 1](#_Toc119163768)

[1. General information 2](#_Toc119163769)

[2. Experimental procedures 3](#_Toc119163770)

[3. The in vitro antitumor assay 7](#_Toc119163771)

[3. Characterization of compounds 8](#_Toc119163772)

[4. NMR copies of product 26](#_Toc119163773)

[6. References 66](#_Toc119163774)

## 1. General information

#### Cesium carbonate (Cs_2_CO_3_) was purchased from Damasbeta, Shanghai, China. Other reagents were purchased from energy-chemical.com. Unless otherwise stated, all commercially available reagents were directly used without further purification. All solvents were purified by standard methods prior to use. All reactions were monitored by thin layer chromatography (TLC), and column chromatography was carried out on 200-300 mesh of silica gel purchased from Tansoole, Shanghai, China. All nuclear magnetic resonance (NMR) spectra were recorded on a Bruker Avance 400 MHz or 600 MHz in CDCl_3_ at room temperature (20 ± 3 °C), using tetramethylsilane as internal standard. High resolution mass spectra (HRMS) were conducted on a 3000-mass spectrometer, using Bruker compact Qq TOF MS/MS system with the ESI technique.

Photochemical reaction was carried out under visible light irradiation by a purple LED at 25 ºC. OHSP - 350UV 8-position Photo Reaction System manufactured by Beijing Roger Tech Ltd. was used in this system (See Figure S1). Eight 10W purple LEDs were equipped in this Photo reactor. The purple LED's energy peak wavelength is 405 nm, peak width at half-height is 13.6 nm, lirradiance@5 W is 87.93 mW/cm^2^. The reaction vessel is borosilicate glass test tube and no filters were applied.


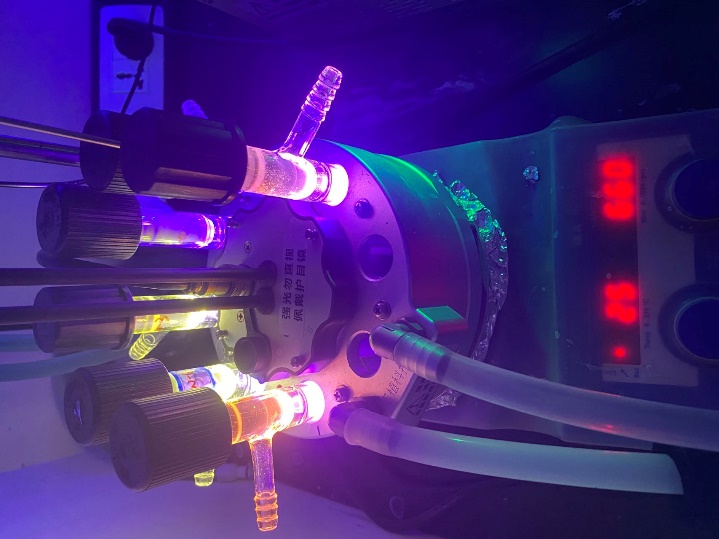

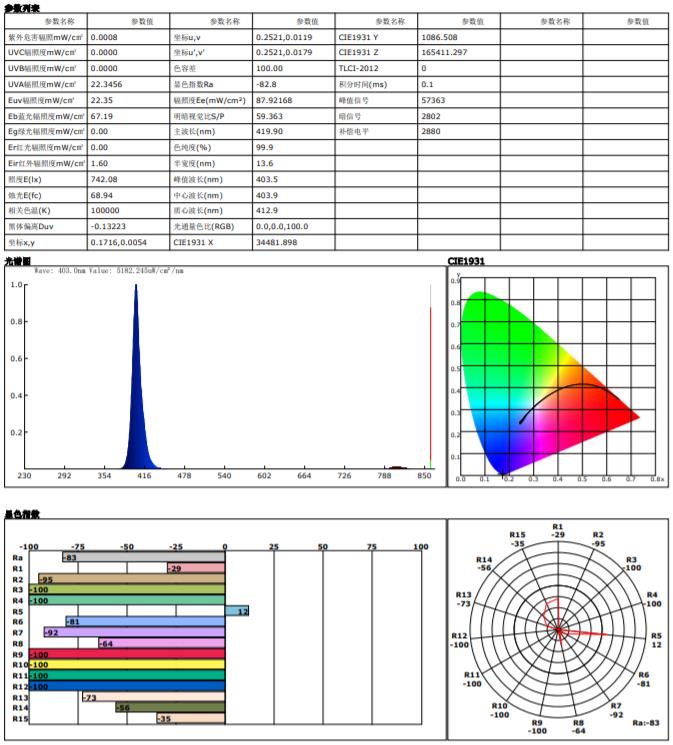


**Figure S1.** The reaction apparatus and spectrum of 405 nm LED

## Experimental procedures

**2.1 General experimental procedures for 3-alkylated 2*H*-indazoles**

In a 10 mL reaction vial with a stirring bar, 2-phenyl-2*H*-indazole **1** (0.2 mmol), 2-(hexylamino)-2-oxoacetic acid **2** (2.5 equiv.), Cs_2_CO_3_ (2.0 equiv.) and 4CzIPN (5 mol%) were added. The vial was then evacuated and backfilled three times with O_2_, followed by adding dimethyl sulfoxide (3 mL). The mixture was stirred at room temperature with 10 W 405nm LED irradiation for 12 h. After the reaction was completed, it was quenched with water (15 mL), and then the ethyl acetate (15 mL) was added three times for extraction. The combined organic layers were dried over anhydrous Na_2_SO_4_ and evaporated under vacuum. The residue was purified by silica gel chromatography (petroleum ether/ethyl acetate = 7/1) to afford the desired product **3**.

**2.2 Control experiments**

**Control experiments with TEMPO:** In a 10 mL reaction vial with a stirring bar, 2-phenyl-2*H*-indazole **1a** (0.2 mmol), 2-(hexylamino)-2-oxoacetic acid **2a** (2.5 equiv.), Cs_2_CO_3_ (2.0 equiv.), 4CzIPN (5 mol%) and 2,2,6,6-tetramethyl-1-piperidinyloxy (TEMPO, 3.0 equiv.) were added. The vial was then evacuated and backfilled three times with O_2_, followed by adding Dimethyl sulfoxide (3 mL). The mixture was stirred at room temperature with 10 W 405nm LED irradiation for 12 h. After the reaction was completed, it was quenched with water (15 mL), and then the ethyl acetate (15 mL) was added three times for extraction. No target product **3a** was generated, while the formation of TEMPO trapped cyclohexyl adduct **5a** was found by HRMS. It indicated that a radical pathway should be involved in this photocatalytic reaction and an amide radical was formed.

**Control experiments with BHT:** In a 10 mL reaction vial with a stirring bar, 2-phenyl-2*H*-indazole **1** (0.2 mmol), 2-(hexylamino)-2-oxoacetic acid **2** (2.5 equiv.), Cs_2_CO_3_ (2.0 equiv.), 4CzIPN (5 mol%) and 2,6-di-*tert*-butyl-4-methyl phenol (BHT, 3.0 equiv) were added. The mixture was stirred at room temperature with 10 W 405nm LED irradiation for 12 h. After the reaction was completed, it was quenched with water (15 mL), and then the ethyl acetate (15 mL) was added three times for extraction.. The combined organic layers were dried over anhydrous Na_2_SO_4_. The residue was purified by silica gel chromatography (petroleum ether/ethyl acetate = 7/1) to afford the desired product **3a** in 25% yield.

**2.3 Procedure for emission quenching experiment**

Stern-Volmer fluorescence quenching experiments were conducted via adding the appropriate amount of **1a** and **6** to a freshly prepared solution of 4CzIPN (1×10^-4^ M) in dry DMSO in a screw-top quartz cuvette at room temperature. After degassing with a stream of N_2_ for 10 minutes, the sample was irradiated at 405 nm and the fluorescence was measured from 380 nm to 800 nm (Figure S2).

Figure S2. (A) The emission spectra of 1×10^-4^ M solution of 4CzIPN with 8 mM of **1a** and **6.** (B) The emission spectra of 1×10^-4^ M solution of 4CzIPN with various concentrations of **1a**. (C) The emission spectra of 1×10^-4^ M solution of 4CzIPN with various concentrations of **6**. (D) The linear relationship between I_0_/I (I_0_ and I are the fluorescence intensities before and after adding the different components of various concentrations, respectively) and the concentration.

**2.4 Procedure for cyclic voltametric experiment**

Cyclic voltammetry analysis of the cesium salt of **2a** (**6**) were conducted by a potentiostat (CH instrument, 660E) with a three electrode system (Reference electrode: SCE, working electrode: Glassy carbon, counter electrode: Pt wire). 0.1 M Bu_4_NPF_6_ in CH_3_CN was used as supporting electrolyte. The Pt disk was polished by using an alumina suspension (*d* = 50 nm) before each CV experiments (Figure S3).

Figure S3. CV of **6** (5 mM in CH_3_CN) and blank (only 0.1 M Bu_4_NPF_6_) under nitrogen atmosphere at room temperature. The scan rate was 0.10 V/s, ranging from 0 V to 2 V.

## 3. The in vitro antitumor assay

The *in vitro* antitumor assay against Ramos cell was evaluated by the CellTiter-Glo (Promega, USA) assay. Ramos cell suspensions were diluted in a growth medium to desired density and added 95 µL to a 96-well plate. 5 µL of the tested compounds were added to the 96-well plate. Then the cell was incubated at 37 ℃, 5% CO_2_ for 72 h. Equilibrate the assay plate to room temperature before measurement. Add 20 µL of CellTiter-Glo^®^ Reagent into each well. Mix contents for 2 minutes on an orbital shaker to induce cell lysis. Incubate at room temperature for 10 minutes to stabilize luminescent signal. Record luminescence using EnVision Multilabel Reader (PerkinElmer). Cell viability (CV%) was calculated relative to vehicle (DMSO) treated control wells using following formula: Cell viability (%) = (RLU compound - RLU blank) / (RLU control - RLU blank) * 100%. The IC_50_ values were calculated using GraphPad Prism 6.0 software, fitting to a 4-parameter equation to generate concentration-response curves.

## Characterization of compounds

*N-hexyl-2-phenyl-2H-indazole-3-carboxamide (****3a****)*

58.8 mg, 91%; White solid, m.p. 135‑136 °C; ^1^H NMR (400 MHz, CDCl_3_) δ 7.92 (d, *J* = 8.8 Hz, 1H), 7.80 (d, *J* = 8.8 Hz, 1H), 7.62 ‒ 7.59 (m, 2H), 7.56 ‒ 7.51 (m, 3H), 7.40 ‒ 7.36 (m, 1H), 7.28 ‒ 7.24 (m, 1H), 5.79 (s, 1H), 3.42 – 3.37 (m, 2H), 1.52 ‒ 1.45 (m, 2H), 1.31 – 1.26 (m, 6H), 0.88 (t, *J* = 6.8 Hz, 3H). ^13^C NMR (100 MHz, CDCl_3_) δ 159.7, 148.6, 140.3, 129.6, 129.4, 127.3, 125.9, 124.6, 122.6, 120.3, 118.5, 40.0, 31.5, 29.5, 26.7, 22.7, 14.1. HRMS (ESI-TOF) m/z: Calcd for C_20_H_24_N_3_O [M + H]^+^ 322.1914, Found: 322.1916.

*N-hexyl-2-(p-tolyl)-2H-indazole-3-carboxamide (****3b****)*

48.0 mg, 72%; White solid, m.p. 146-147 °C; ^1^H NMR (400 MHz, CDCl_3_) δ 7.93 (d, *J* = 8.4 Hz, 1H), 7.78 (d, *J* = 8.8 Hz, 1H), 7.47 (d, *J* = 8.4 Hz, 2H), 7.39 ‒ 7.31 (m, 3H), 7.26 ‒ 7.23 (m, 1H), 5.80 (s, 1H), 3.40 – 3.36 (m, 2H), 2.45 (s, 3H), 1.51 – 1.44 (m, 2H), 1.29 – 1.23 (m, 6H), 0.89 (t, *J*= 6.4 Hz, 3H). ^13^C NMR (100 MHz, CDCl_3_) δ 159.7, 148.5, 139.8, 137.8, 130.0, 129.3, 127.1, 125.7, 124.5, 122.7, 120.4, 118.4, 39.9, 31.6, 29.5, 26.7, 22.7, 21.4, 14.1. HRMS (ESI-TOF) m/z: Calcd for C_21_H_26_N_3_O [M + H]^+^ 336.2070, Found: 336.2064.

*N-hexyl-2-(m-tolyl)-2H-indazole-3-carboxamide (****3c****)*

43.1 mg, 64%; Yellow solid, m.p. 115-116 °C; ^1^H NMR (400 MHz, CDCl_3_) δ 7.95 (d, *J* = 8.4 Hz, 1H), 7.78 (d, *J* = 8.8 Hz, 1H), 7.43 – 7.31 (m, 5H), 7.25 ‒ 7.23 (m, 1H), 5.77 (s, 1H), 3.40 – 3.35 (m, 2H), 2.44 (s, 3H), 1.49 – 1.42 (m, 2H), 1.29 – 1.22 (m, 6H), 0.88 (t, *J* = 6.8 Hz, 3H). ^13^C NMR (150 MHz, CDCl_3_) δ 159.7, 148.5, 140.1, 139.7, 130.5, 129.4, 129.2, 127.2, 126.5, 124.5, 123.0, 122.8, 120.5, 118.4, 39.9, 31.6, 29.5, 26.6, 22.7, 21.5, 14.1. HRMS (ESI-TOF) m/z: Calcd for C_21_H_26_N_3_O [M + H]^+^ 336.2070, Found: 336.2061.

*2-(4-chlorophenyl)-N-hexyl-2H-indazole-3-carboxamide (****3d****)*

41.0 mg, 58%; White solid, m.p. 170-171 °C; ^1^H NMR (400 MHz, CDCl_3_) δ 7.82 – 7.79 (m, 2H), 7.56 – 7.53 (m, 2H), 7.50 ‒ 7.47 (m, 2H), 7.41 ‒ 7.37 (m, 1H), 7.29 ‒ 7.26 (m, 1H), 5.98 (s, 1H), 3.47 – 3.42 (m, 2H), 1.60 – 1.54 (m, 2H), 1.34 – 1.31 (m, 6H), 0.92 – 0.88 (m, 3H). ^13^C NMR (150 MHz, CDCl_3_) δ 159.6, 148.7, 138.9, 135.4, 129.39, 129.35, 127.4, 127.0, 124.8, 122.0, 119.7, 118.7, 40.1, 31.6, 29.6, 26.8, 22.7, 14.1. HRMS (ESI-TOF) m/z: Calcd for C_20_H_23_ClN_3_O [M + H]^+^ 356.1524, Found: 356.1522.

*2-(3-chlorophenyl)-N-hexyl-2H-indazole-3-carboxamide (****3e****)*

60.0 mg, 84%; White solid, m.p. 153‑154 °C; ^1^H NMR (600 MHz, CDCl_3_) δ 7.83 – 7.79 (m, 2H), 7.65 (t, *J* = 1.8 Hz, 1H), 7.51 ‒ 7.43 (m, 3H), 7.40 ‒ 7.38 (m, 1H), 7.28 ‒ 7.27 (m, 1H), 5.97 (s, 1H), 3.47 – 3.43 (m, 2H), 1.59 – 1.55 (m, 2H), 1.33 – 1.31 (m, 6H), 0.90 (t, *J* = 6.6 Hz, 3H). ^13^C NMR (150 MHz, CDCl_3_) δ 159.6, 148.7, 141.3, 135.0, 130.1, 129.6, 129.5, 127.5, 126.2, 124.9, 124.0, 122.1, 119.8, 118.7, 40.1, 31.6, 29.6, 26.7, 22.7, 14.2. HRMS (ESI-TOF) m/z: Calcd for C_20_H_23_ClN_3_O [M + H]^+^ 356.1524, Found: 356.1512.

*2-(4-bromophenyl)-N-hexyl-2H-indazole-3-carboxamide (****3f****)*

39.1 mg, 49%; White solid, m.p. 166-167 °C; ^1^H NMR (600 MHz, CDCl_3_) δ 7.81 (t, *J* = 7.8 Hz, 2H), 7.65 ‒ 7.64 (m, 2H), 7.50 ‒ 7.49 (m, 2H), 7.41 ‒ 7.38 (m, 1H), 7.29 ‒ 7.27 (m, 1H), 5.96 (s, 1H), 3.47 – 3.44 (m, 2H), 1.58 – 1.56 (m, 2H), 1.32 – 1.31 (m, 6H), 0.90 (t, *J* = 6.6 Hz, 3H). ^13^C NMR (150 MHz, CDCl_3_) δ 159.7, 148.7, 139.4, 132.4, 129.3, 127.4, 127.3, 124.9, 123.5, 122.1, 119.6, 118.7, 40.2, 31.5, 29.7, 26.8, 22.7, 14.2. HRMS (ESI-TOF) m/z: Calcd for C_20_H_23_BrN_3_O [M + H]^+^ 400.1019, Found: 400.1083.

*2-(3-bromophenyl)-N-hexyl-2H-indazole-3-carboxamide (****3g****)*

41.9 mg, 52%; White solid, m.p. 164-165 °C; ^1^H NMR (600 MHz, CDCl_3_) δ 7.82 – 7.78 (m, 3H), 7.63 – 7.61 (m, 1H), 7.55 – 7.53 (m, 1H), 7.40 ‒ 7.36 (m, 2H), 7.27 ‒ 7.25 (m, 1H), 5.99 (s, 1H), 3.46 – 3.42 (m, 2H), 1.58 ‒ 1.55 (m, 2H), 1.33 – 1.31 (m, 6H), 0.90 (t, *J* = 6.6 Hz, 3H). ^13^C NMR (150 MHz, CDCl_3_) δ 159.6, 148.7, 141.3, 132.5, 130.4, 129.5, 128.9, 127.5, 124.9, 124.5, 122.7, 122.1, 119.8, 118.6, 40.1, 31.6, 29.6, 26.7, 22.7, 14.2. HRMS (ESI-TOF) m/z: Calcd for C_20_H_23_BrN_3_O [M + H]^+^ 400.1019, Found: 400.1004.

*N-hexyl-2-(4-(trifluoromethyl)phenyl)-2H-indazole-3-carboxamide (****3h****)*

62.8 mg, 81%; White solid, m.p. 152‑153 °C; ^1^H NMR (600 MHz, CDCl_3_) δ 7.81 (d, *J* = 8.4 Hz, 1H), 7.78 – 7.74 (m, 5H), 7.42 ‒ 7.39 (m, 1H), 7.30 ‒ 7.27 (m, 1H), 6.10 (s, 1H), 3.49 – 3.45 (m, 2H), 1.63 – 1.59 (m, 2H), 1.37 – 1.31 (m, 6H), 0.90 (t, *J* = 6.6 Hz, 3H). ^13^C NMR (150 MHz, CDCl_3_) δ 159.6, 148.9, 143.2, 131.2 (q, *J* = 33 Hz), 129.5, 127.6, 126.3 (q, *J* = 3.0 Hz), 126.1, 125.1, 123.8 (q, *J* = 271.5 Hz), 121.9, 119.4, 118.9, 40.2, 31.5, 29.7, 26.8, 22.7, 14.1. ^19^F NMR (564 MHz, CDCl_3_) δ -62.6. HRMS (ESI-TOF) m/z: Calcd for C_21_H_23_F_3_N_3_O [M + H]^+^ 390.1788, Found: 390.1766.

*N-hexyl-5-methoxy-2-phenyl-2H-indazole-3-carboxamide (****3i****)*

54.0 mg, 77%; White solid, m.p. 125‑126 °C; ^1^H NMR (600 MHz, CDCl_3_) δ 7.67 (d, *J* = 9.0 Hz, 1H), 7.60 – 7.59 (m, 2H), 7.57 – 7.52 (m, 3H), 7.27 (d, *J* = 2.4 Hz, 1H), 7.07 (dd, *J*_1_ = 9.6 Hz, *J*_2_ = 2.4 Hz, 1H), 5.48 (s, 1H), 3.89 (s, 3H), 3.36 – 3.33 (m, 2H), 1.42 – 1.37 (m, 2H), 1.29 – 1.15 (m, 6H), 0.88 (t, *J* = 7.2 Hz, 3H). ^13^C NMR (150 MHz, CDCl_3_) δ 160.0, 157.4, 145.6, 140.2, 129.7, 129.6, 128.3, 126.1, 124.2, 122.7, 119.7, 97.0, 55.7, 39.8, 31.5, 29.4, 26.7, 22.7, 14.2. HRMS (ESI-TOF) m/z: Calcd for C_21_H_26_N_3_O_2_ [M + H]^+^ 352.2020, Found: 352.2022.

*5-fluoro-N-hexyl-2-phenyl-2H-indazole-3-carboxamide (****3j****)*

47.5 mg, 70%; White solid, m.p. 152‑153 °C; ^1^H NMR (600 MHz, CDCl_3_) δ 7.76 – 7.74 (m, 1H), 7.59 – 7.57 (m, 2H), 7.55 ‒ 7.53 (m, 4H), 7.18 ‒ 7.15 (m, 1H), 5.70 (s, 1H), 3.36 – 3.32 (m, 2H), 1.45 – 1.41 (m, 2H), 1.28 – 1.19 (m, 6H), 0.88 (t, *J* = 7.2 Hz, 3H). ^13^C NMR (150 MHz, CDCl_3_) δ 160.0 (d, *J* = 241.5 Hz), 159.4, 146.0, 140.0, 129.9, 129.7 (d, *J* = 9.0 Hz), 129.5, 125.9, 122.7 (d, *J* = 12.0 Hz), 120.6 (d, *J* = 9.0 Hz), 119.0 (d, *J* = 28.5 Hz), 103.5 (d, *J* = 25.5 Hz), 39.9, 31.5, 29.4, 26.6, 22.6, 14.1. ^19^F NMR (564 MHz, CDCl_3_) δ -115.7. HRMS (ESI-TOF) m/z: Calcd for C_20_H_23_FN_3_O [M + H]^+^ 340.1820, Found: 340.1800.

*5-chloro-N-hexyl-2-phenyl-2H-indazole-3-carboxamide (****3k****)*

53.0 mg, 74%; White solid, m.p. 161‑162 °C; ^1^H NMR (600 MHz, CDCl_3_) δ 7.96 (d, *J* = 1.2 Hz, 1H), 7.72 (d, *J* = 9.0 Hz, 1H), 7.60 ‒ 7.59 (m, 2H), 7.56 ‒ 7.54 (m, 3H), 7.31 (dd, *J*_1_ = 9.0 Hz, *J*_2_ = 1.8 Hz, 1H), 5.67 (s, 1H), 3.38 – 3.35 (m, 2H), 1.47 – 1.43 (m, 2H), 1.30 – 1.20 (m, 6H), 0.89 (t, *J* = 7.2 Hz, 3H). ^13^C NMR (150 MHz, CDCl_3_) δ 159.2, 146.9, 139.9, 130.4, 129.9, 129.5, 129.1, 128.8, 125.8, 123.2, 119.8, 119.4, 40.0, 31.5, 29.4, 26.6, 22.6, 14.1. HRMS (ESI-TOF) m/z: Calcd for C_20_H_23_ClN_3_O [M + H]^+^ 356.1524, Found: 356.1531.

*5-bromo-N-hexyl-2-phenyl-2H-indazole-3-carboxamide (****3l****)*

38.9 mg, 49%; White solid, m.p. 156‑157 °C; ^1^H NMR (600 MHz, CDCl_3_) δ 8.17 (d, *J* = 1.2 Hz, 1H), 7.67 (d, *J* = 9.0 Hz, 1H), 7.61 ‒ 7.59 (m, 2H), 7.57 ‒ 7.55 (m, 3H), 7.44 (dd, *J*_1_ = 9.6 Hz, *J*_2_ = 1.8 Hz, 1H), 5.65 (s, 1H), 3.38 – 3.35 (m, 2H), 1.48 – 1.43 (m, 2H), 1.30 – 1.21 (m, 6H), 0.89 (t, *J* = 7.2 Hz, 3H). ^13^C NMR (150 MHz, CDCl_3_) δ 159.2, 147.0, 139.9, 131.2, 130.0, 129.6, 129.0, 125.9, 124.0, 122.9, 120.1, 118.4, 40.0, 31.5, 29.4, 26.7, 22.7, 14.2. HRMS (ESI-TOF) m/z: Calcd for C_20_H_23_BrN_3_O [M + H]^+^ 400.1019, Found: 400.0991.

5-chloro-N-hexyl-2-(p-tolyl)-2H-indazole-3-carboxamide *(****3m****)*

38.0 mg, 51%; White solid, m.p. 157‑158 °C; ^1^H NMR (400 MHz, CDCl_3_) δ 7.98 (d, *J* = 1.2 Hz, 1H), 7.71 (d, *J* = 9.2 Hz, 1H), 7.46 (d, *J* = 8.4 Hz, 2H), 7.35 ‒ 7.29 (m, 3H), 5.66 (s, 1H), 3.38 – 3.33 (m, 2H), 2.46 (s, 3H), 1.48 – 1.41 (m, 2H), 1.31 – 1.19 (m, 6H), 0.89 (t, *J* = 6.8 Hz, 3H). ^13^C NMR (150 MHz, CDCl_3_) δ 159.3, 146.8, 140.3, 137.4, 130.3, 130.2, 129.1, 128.7, 125.7, 123.4, 119.8, 119.6, 39.9, 31.5, 29.4, 26.7, 22.7, 21.4, 14.1. HRMS (ESI-TOF) m/z: Calcd for C_21_H_25_ClN_3_O [M + H]^+^ 370.1681, Found: 370.1698.

5-fluoro-N-hexyl-2-(p-tolyl)-2H-indazole-3-carboxamide *(****3n****)*

42.0 mg, 59%; White solid, m.p. 162‑163 °C; ^1^H NMR (600 MHz, CDCl_3_) δ 7.76 – 7.74 (m, 1H), 7.59 – 7.57 (m, 1H), 7.46 (d, *J* = 8.4 Hz, 2H), 7.34 (d, *J* = 7.8 Hz, 2H), 7.18 – 7.15 (m, 1H), 5.65 (s, 1H), 3.36 – 3.33 (m, 2H), 2.46 (s, 3H), 1.46 – 1.41 (m, 2H), 1.30 – 1.17 (m, 6H), 0.88 (t, *J* = 7.2 Hz, 3H). ^13^C NMR (150 MHz, CDCl_3_) δ 160.0 (d, *J* = 243.0 Hz), 159.4, 145.9, 140.2, 137.6, 130.1, 129.6 (d, *J* = 9.0 Hz), 125.8, 122.8 (d, *J* = 12.0 Hz), 120.5 (d, *J* = 9.0 Hz), 118.9 (d, *J* = 28.5 Hz), 103.6 (d, *J* = 25.5 Hz), 39.9, 31.5, 29.4, 26.6, 22.7, 21.4, 14.1. ^19^F NMR (564 MHz, CDCl_3_) δ -115.9. HRMS (ESI-TOF) m/z: Calcd for C_21_H_25_FN_3_O [M + H]^+^ 354.1976, Found: 354.1958.

2-(4-chlorophenyl)-N-hexyl-5-methoxy-2H-indazole-3-carboxamide *(****3o****)*

38.0 mg, 49%; White solid, m.p. 182‑183 °C; ^1^H NMR (400 MHz, CDCl_3_) δ 7.66 – 7.64 (m, 1H), 7.53 – 7.46 (m, 4H), 7.08 – 7.05 (m, 2H), 5.73 (s, 1H), 3.88 (s, 3H), 3.42 – 3.37 (m, 2H), 1.54 – 1.46 (m, 2H), 1.30 – 1.25 (m, 6H), 0.89 (t, *J* = 6.4 Hz, 3H). ^13^C NMR (100 MHz, CDCl_3_) δ 160.0, 157.4, 145.7, 138.8, 135.4, 129.5, 128.3, 127.0, 123.4, 122.8, 119.8, 96.3, 55.6, 39.9, 31.6, 29.5, 26.8, 22.7, 14.1. HRMS (ESI-TOF) m/z: Calcd for C_21_H_25_ClN_3_O_2_ [M + H]^+^ 386.1630, Found: 386.1620.

*N-butyl-2-phenyl-2H-indazole-3-carboxamide (****3p****)*

55.0 mg, 94%; White solid, m.p. 148-149 °C; ^1^H NMR (400 MHz, CDCl_3_) δ 7.92 (d, *J* = 8.8 Hz, 1H), 7.80 (d, *J* = 8.8 Hz, 1H), 7.61 ‒ 7.58 (m, 2H), 7.56 – 7.51 (m, 3H), 7.40 ‒ 7.36 (m, 1H), 7.28 – 7.24 (m, 1H), 5.80 (s, 1H), 3.42 – 3.37 (m, 2H), 1.51 – 1.44 (m, 2H), 1.32 – 1.23 (m, 2H), 0.90 (t, *J* = 7.2 Hz, 3H). ^13^C NMR (150 MHz, CDCl_3_) δ 159.7, 148.6, 140.3, 129.6, 129.4, 127.3, 125.9, 124.6, 122.6, 120.3, 118.5, 39.7, 31.6, 20.1, 13.8. HRMS (ESI-TOF) m/z: Calcd for C_18_H_20_N_3_O [M + H]^+^ 294.1601, Found: 294.1608.

*methyl 5-(2-phenyl-2H-indazole-3-carboxamido)pentanoate (****3q****)*

41.0 mg, 58%; White solid, m.p. 128-129°C; ^1^H NMR (600 MHz, CDCl_3_) δ 7.92 (d, *J* = 8.4 Hz, 1H), 7.79 (d, *J* = 9.0 Hz, 1H), 7.60 – 7.58 (m, 2H), 7.54 – 7.49 (m, 3H), 7.39 – 7.36 (m, 1H), 7.27 – 7.25 (m, 1H), 5.98 (s, 1H), 3.66 (s, 3H), 3.42 – 3.38 (m, 2H), 2.33 – 2.30 (m, 2H), 1.61 – 1.52 (m, 4H). ^13^C NMR (150 MHz, CDCl_3_) δ 173.9, 159.8, 148.6, 140.3, 129.6, 129.4, 129.2, 127.2, 125.9, 124.6, 122.5, 120.2, 118.5, 51.8, 39.4, 33.5, 29.0, 22.1. HRMS (ESI-TOF) m/z: Calcd for C_20_H_22_N_3_O_3_ [M + H]^+^ 352.1656, Found: 352.1648.

*methyl 6-(2-phenyl-2H-indazole-3-carboxamido)hexanoate (****3r****)*

52.0 mg, 71%; Yellow solid, m.p. 80-81°C; ^1^H NMR (600 MHz, CDCl_3_) δ 7.91 (d, *J* = 8.4 Hz, 1H), 7.79 (d, *J* = 9.0 Hz, 1H), 7.60 – 7.59 (m, 2H), 7.55 – 7.50 (m, 3H), 7.39 – 7.37 (m, 1H), 7.27 – 7.25 (m, 1H), 5.87 (s, 1H), 3.65 (s, 3H), 3.41 – 3.38 (m, 2H), 2.29 (t, *J* = 7.2 Hz, 2H), 1.63 – 1.59 (m, 2H), 1.52 – 1.49 (m, 2H), 1.31 – 1.27 (m, 2H). ^13^C NMR (150 MHz, CDCl_3_) δ 174.0, 159.7, 148.6, 140.3, 129.6, 129.4, 129.3, 127.3, 125.9, 124.6, 122.6, 120.2, 118.5, 51.7, 39.6, 33.9, 29.2, 26.4, 24.5. HRMS (ESI-TOF) m/z: Calcd for C_21_H_24_N_3_O_3_ [M + H]^+^ 366.1812, Found: 336.1808.

*methyl 8-(2-phenyl-2H-indazole-3-carboxamido)octanoate (****3s****)*

49.0 mg, 62%; White solid, m.p. 88-89°C; ^1^H NMR (600 MHz, CDCl_3_) δ 7.93 (d, *J* = 9.0 Hz, 1H), 7.80 (d, *J* = 8.4 Hz, 1H), 7.62 – 7.60 (m, 2H), 7.56 – 7.51 (m, 3H), 7.40 – 7.37 (m, 1H), 7.28 (d, *J* = 7.8 Hz, 1H), 5.78 (s, 1H), 3.66 (s, 3H), 3.41 – 3.37 (m, 2H), 2.31 – 2.29 (m, 2H), 1.62 – 1.60 (m, 2H), 1.51 – 1.46 (m, 2H), 1.31 – 1.24 (m, 6H). ^13^C NMR (100 MHz, CDCl_3_) δ 174.3, 159.7, 148.6, 140.3, 129.7, 129.41, 129.36, 127.3, 126.0, 124.6, 122.6, 120.3, 118.5, 51.6, 39.9, 34.1, 29.5, 29.1, 29.0, 26.8, 24.9. HRMS (ESI-TOF) m/z: Calcd for C_23_H_28_N_3_O_3_ [M + H]^+^ 394.2125, Found: 394.2107.

*2-phenyl-3-(tetrahydro-2H-pyran-4-yl)-2H-indazole (****3t****)*^1^

27.2 mg, 42%; White solid, m.p. 181‑182 °C; ^1^H NMR (600 MHz, CDCl_3_) δ 7.89 (d, *J* = 8.4 Hz, 1H), 7.80 (d, *J* = 8.4 Hz, 1H), 7.56 ‒ 7.55 (m, 2H), 7.48 – 7.47 (m, 3H), 7.39 – 7.36 (m, 1H), 7.34 – 7.29 (m, 3H), 7.25 – 7.21 (m, 3H), 6.12 (s, 1H), 4.58 (d, *J* = 6.0 Hz, 2H). ^13^C NMR (150 MHz, CDCl_3_) δ 159.6, 148.6, 140.2, 137.6, 129.6, 129.4, 129.0, 128.0, 127.9, 127.3, 125.9, 124.8, 122.7, 120.2, 118.5, 44.1. HRMS (ESI-TOF) m/z: Calcd for C_21_H_18_N_3_O [M + H]^+^ 328.1444, Found: 328.1441.

*N-cyclopentyl-2-phenyl-2H-indazole-3-carboxamide (****3u****)*^1^

49.0 mg, 80%; White solid, m.p. 180‑181 °C; ^1^H NMR (600 MHz, CDCl_3_) δ 7.93 (d, *J* = 8.4 Hz, 1H), 7.79 (d, *J* = 8.4 Hz, 1H), 7.60 ‒ 7.59 (m, 2H), 7.55 ‒ 7.51 (m, 3H), 7.39 – 7.36 (m, 1H), 7.25 – 7.24 (m, 1H), 5.69 (s, 1H), 4.37 – 4.32 (m, 1H), 1.98 – 1.95 (m, 2H), 1.60 – 1.51 (m, 4H), 1.35 – 1.30 (m, 2H). ^13^C NMR (150 MHz, CDCl_3_) δ 159.2, 148.6, 140.2, 129.7, 129.5, 129.4, 127.3, 126.0, 124.5, 122.7, 120.4, 118.4, 51.7, 33.1, 23.7. HRMS (ESI-TOF) m/z: Calcd for C_19_H_20_N_3_O [M + H]^+^ 306.1601, Found: 306.1587.

*N-cyclohexyl-2-phenyl-2H-indazole-3-carboxamide (****3v****)*^1^

50.8 mg, 80%; White solid, m.p. 211‑212°C; ^1^H NMR (600 MHz, CDCl_3_) δ 7.91 (d, *J* = 8.4 Hz, 1H), 7.78 (d, *J* = 8.4 Hz, 1H), 7.60 ‒ 7.58 (m, 2H), 7.54 – 7.51 (m, 3H), 7.38 ‒ 7.35 (m, 1H), 7.25 – 7.23 (m, 1H), 5.70 (d, *J* = 7.2 Hz, 1H), 3.97 – 3.91 (m, 1H), 1.92 – 1.89 (m, 2H), 1.63 – 1.57 (m, 3H), 1.39 – 1.33 (m, 2H), 1.18 – 1.05 (m, 3H). ^13^C NMR (150 MHz, CDCl_3_) δ 158.8, 148.6, 140.2, 129.6, 129.5, 129.4, 127.2, 125.9, 124.5, 122.6, 120.3, 118.4, 48.6, 32.9, 25.5, 24.7. HRMS (ESI-TOF) m/z: Calcd for C_20_H_22_N_3_O [M + H]^+^ 320.1757, Found: 320.1748.

*2-phenyl-N-(2-phenylpropan-2-yl)-2H-indazole-3-carboxamide (****3w****)*

66.0 mg, 93%; White solid, m.p. 156-157 °C; ^1^H NMR (600 MHz, CDCl_3_) δ 7.91 (d, *J* =8.4 Hz, 1H), 7.75 (d, *J* =8.4 Hz, 1H), 7.55 ‒ 7.54 (m, 2H), 7.49 – 7.47 (m, 3H), 7.35 – 7.32 (m, 1H), 7.26 (d, *J* = 4.2 Hz, 4H), 7.22 – 7.18 (m, 2H), 6.01 (s, 1H), 1.67 (s, 6H). ^13^C NMR (150 MHz, CDCl_3_) δ 158.7, 156.0, 148.5, 146.1, 140.1, 130.0, 129.7, 129.6, 129.5, 128.6, 127.4, 127.1, 126.0, 124.8, 124.6, 122.6, 120.44, 120.36, 118.4, 115.5, 57.0, 28.9. HRMS (ESI-TOF) m/z: Calcd for C_23_H_22_N_3_O [M + H]^+^ 356.1757, Found: 356.1751.

*N,2-diphenyl-2H-indazole-3-carboxamide (****3x****)*^1^

58.0 mg, 93%; Light yellow oil; ^1^H NMR (600 MHz, CDCl_3_) δ 8.02 (d, *J* = 8.4 Hz, 1H), 7.85 (d, *J* = 8.4 Hz, 1H), 7.66 ‒ 7.65 (m, 2H), 7.58 ‒ 7.54 (m, 3H), 7.44 – 7.41 (m, 3H), 7.34 – 7.31 (m, 3H), 7.16 – 7.13 (m, 1H). ^13^C NMR (150 MHz, CDCl_3_) δ 148.7, 140.1, 137.3, 129.9, 129.6, 129.3, 129.0, 127.4, 126.1, 125.19, 125.15, 122.9, 120.13, 120.08, 118.8. HRMS (ESI-TOF) m/z: Calcd for C_20_H_16_N_3_O [M + H]^+^ 314.1288, Found: 314.1282.

*2-phenyl-N-(p-tolyl)-2H-indazole-3-carboxamide (****3y****)*^1^

40.0 mg, 61%; White solid, m.p. 218‑219 °C; ^1^H NMR (400 MHz, CDCl_3_) δ 8.01 (d, *J* = 8.8 Hz, 1H), 7.84 (d, *J* = 8.8 Hz, 1H), 7.66 – 7.64 (m, 2H), 7.57 ‒ 7.54 (m, 3H), 7.48 (s, 1H), 7.44 – 7.40 (m, 1H), 7.33 – 7.29 (m, 3H), 7.12 (d, *J* = 8.0 Hz, 2H), 2.32 (s, 3H). ^13^C NMR (150 MHz, CDCl_3_) δ 157.4, 148.7, 140.1, 134.9, 134.8, 129.83, 129.79, 129.6, 129.1, 127.4, 126.0, 125.1, 122.9, 120.2, 120.1, 118.7, 21.0. HRMS (ESI-TOF) m/z: Calcd for C_21_H_17_N_3_NaO [M + Na]^+^ 350.1264, Found: 350.1286.

*N-(4-methoxyphenyl)-2-phenyl-2H-indazole-3-carboxamide (****3z****)*^1^

37.1 mg, 54%; Brown solid, m.p. 185-186 °C; ^1^H NMR (600 MHz, CDCl_3_) δ 8.02 (d, *J* = 8.4 Hz, 1H), 7.85 (d, *J* = 9.0 Hz, 1H), 7.66 (d, *J* = 6.6 Hz, 2H), 7.57 – 7.55 (m, 3H), 7.44 – 7.41 (m, 2H), 7.36 – 7.30 (m, 3H), 6.86 (d, *J* = 7.8 Hz, 2H), 3.79 (s, 3H). ^13^C NMR (150 MHz, CDCl_3_) δ 157.4, 157.1, 148.7, 140.2, 130.4, 129.9, 129.6, 129.1, 127.4, 126.1, 125.1, 122.9, 121.9, 120.2, 118.7, 114.5, 55.7. HRMS (ESI-TOF) m/z: Calcd for C_21_H_18_N_3_O_2_ [M + H]^+^ 344.1394, Found: 344.1386.

*N-(4-chlorophenyl)-2-phenyl-2H-indazole-3-carboxamide (****3aa****)*^1^

38.0 mg, 55%; Light yellow oil; ^1^H NMR (600 MHz, CDCl_3_) δ 8.03 (d, *J* = 8.4 Hz, 1H), 7.87 (d, *J* = 8.4 Hz, 1H), 7.67 ‒ 7.66 (m, 2H), 7.60 ‒ 7.58 (m, 3H), 7.46 ‒ 7.43 (m, 2H), 7.38 ‒ 7.34 (m, 3H), 7.29 (d, *J* = 8.4 Hz, 2H). ^13^C NMR (150 MHz, CDCl_3_) δ 148.7, 140.1, 135.9, 130.2, 130.1, 129.7, 129.4, 128.6, 127.5, 126.2, 125.4, 123.1, 121.2, 120.1, 118.9. HRMS (ESI-TOF) m/z: Calcd for C_20_H_15_ClN_3_O [M + H]^+^ 348.0898, Found: 348.0860.

*N,N-dibutyl-2-phenyl-2H-indazole-3-carboxamide (****3ab****)*

32.9 mg, 47%; Light yellow oil; ^1^H NMR (600 MHz, CDCl_3_) δ 7.77 (d, *J* = 9.0 Hz, 1H), 7.75 ‒ 7.73 (m, 2H), 7.61 (d, *J* = 8.4 Hz, 1H), 7.51 – 7.48 (m, 2H), 7.46 – 7.43 (m, 1H), 7.36 – 7.33 (m, 1H), 7.17 -7.15 (m, 1H), 3.62 (s, 1H), 3.36 (s, 1H), 2.90 (s, 2H), 1.65 (s, 1H), 1.54 (s, 2H), 1.31 – 1.25 (m, 3H), 0.95 – 0.93 (m, 5H), 0.62 – 0.60 (m, 3H). ^13^C NMR (150 MHz, CDCl_3_) δ 162.4, 148.8, 140.2, 130.1, 129.4, 129.0, 127.3, 124.4, 123.4, 121.8, 119.8, 118.0, 48.4, 44.7, 30.4, 29.2, 20.4, 19.8, 14.0, 13.6. HRMS (ESI-TOF) m/z: Calcd for C_22_H_28_N_3_O [M + H]^+^ 350.2227, Found: 350.2222.

*(2-phenyl-2H-indazol-3-yl)(piperidin-1-yl)methanone (****3ac****)*^1^

46.0 mg, 75%; White solid, m.p. 130‑131 °C; ^1^H NMR (600 MHz, CDCl_3_) δ 7.78 (d, *J* = 9.0 Hz, 1H), 7.71 (d, *J* = 7.8 Hz, 2H), 7.66 (d, *J* = 8.4 Hz, 1H), 7.53 – 7.50 (m, 2H), 7.47 – 7.44 (m, 1H), 7.37 – 7.34 (m, 1H), 7.19 – 7.16 (m, 1H), 3.83 (s, 1H), 3.60 (s, 1H), 3.10 (s, 1H), 3.04 (s, 1H), 1.65 (s, 1H), 1.54 – 1.50 (m, 3H), 1.34 (s, 1H), 0.89 (s, 1H). ^13^C NMR (150 MHz, CDCl_3_) δ 161.1, 148.9, 140.2, 129.5, 129.4, 129.0, 127.4, 124.4, 123.6, 122.0, 119.9, 118.1, 48.0, 43.2, 26.2, 25.5, 24.4. HRMS (ESI-TOF) m/z: Calcd for C_19_H_20_N_3_O [M + H]^+^ 306.1601, Found: 306.1590.

2-phenyl-2H-indazole-3-carboxamide *(****3ad****)*

26.0 mg, 55%; White solid, m.p. 251‑252 °C; ^1^H NMR (400 MHz, CDCl_3_) δ 8.02 (d, *J* = 8.4 Hz, 1H), 7.83 (d, *J* = 8.8 Hz, 1H), 7.64 ‒ 7.62 (m, 2H), 7.59 ‒ 7.55 (m, 3H), 7.43 – 7.40 (m, 1H), 7.34 – 7.30 (m, 1H), 5.74 (s, 2H). ^13^C NMR (150 MHz, CDCl_3_) δ 161.0, 148.7, 140.2, 129.9, 129.5, 128.1, 127.4, 126.2, 125.2, 123.3, 120.5, 118.7. HRMS (ESI-TOF) m/z: Calcd for C_14_H_12_N_3_O [M + H]^+^ 238.0975, Found: 238.0953.

*N-(6-((R)-2-((1r,4R)-4-isopropylcyclohexane-1-carboxamido)-3-phenylpropanamido)hexyl)-2-phenyl-2H-indazole-3-carboxamide (****4a****)*

79.8 mg, 63%; White solid, m.p. 195-196 °C; ^1^H NMR (600 MHz, CDCl_3_) δ 7.92 (d, *J* = 8.4 Hz, 1H), 7.81 (d, *J* = 9.0 Hz, 1H), 7.62 – 7.61 (m, 2H), 7.55 – 7.50 (m, 3H), 7.40 – 7.38 (m, 1H), 7.29 – 7.27 (m, 3H), 7.23 – 7.17 (m, 3H), 6.08 (d, *J* = 7.2 Hz, 1H), 5.96 – 5.95 (m, 1H), 5.76 – 5.73 (m, 1H), 4.53 – 4.50 (m, 1H), 3.41 – 3.37 (m, 2H), 3.11 (q, *J* = 6.6 Hz, 2H), 3.05 – 2.96 (m, 2H), 1.98 – 1.92 (m, 1H), 1.84 – 1.72 (m, 4H), 1.50 – 1.45 (m, 2H), 1.40 – 1.28 (m, 6H), 1.22 – 1.17 (m, 4H), 1.03 – 0.97 (m, 1H), 0.94 – 0.92 (m, 1H), 0.83 (d, *J* = 6.6 Hz, 6H). ^13^C NMR (150 MHz, CDCl_3_) δ 176.4, 171.0, 159.9, 148.6, 140.3, 137.0, 129.6, 129.4, 128.8, 127.3, 127.1, 125.9, 124.6, 122.6, 120.3, 118.5, 54.5, 51.0, 45.6, 43.3, 39.5, 39.2, 38.5, 32.9, 29.9, 29.6, 29.4, 29.2, 29.1, 29.0, 26.2, 26.0, 19.9. HRMS (ESI-TOF) m/z: Calcd for C_39_H_50_N_5_O_3_ [M + H]^+^ 636.3908, Found: 636.3875.

*N-(6-(5-(2,5-dimethylphenoxy)-2,2-dimethylpentanamido)hexyl)-2-phenyl-2H-indazole-3-carboxamide (****4b****)*

40.0 mg, 35%; Light yellow oil; ^1^H NMR (600 MHz, CDCl_3_) δ 7.92 (d, *J* = 8.4 Hz, 1H), 7.81 (d, *J* = 8.4 Hz, 1H), 7.61 – 7.60 (m, 2H), 7.55 – 7.50 (m, 3H), 7.39 – 7.37 (m, 1H), 7.28 – 7.26 (m, 1H), 6.97 (d, *J* = 7.2 Hz, 1H), 6.63 (d, *J* = 7.2 Hz, 1H), 6.59 (s, 1H), 5.91 (t, *J* = 5.4 Hz, 1H), 5.73 (t, *J* = 5.4 Hz, 1H), 3.89 (t, *J* = 6.0 Hz, 2H), 3.41 – 3.37 (m, 2H), 3.24 – 3.20 (m, 2H), 2.28 (s, 3H), 2.15 (s, 3H), 1.75 – 1.70 (m, 2H), 1.67 – 1.65 (m, 2H), 1.52 – 1.45 (m, 4H), 1.30 – 1.28 (m, 4H), 1.19 (s, 6H). ^13^C NMR (150 MHz, CDCl_3_) δ 177.6, 159.8, 157.0, 148.6, 140.3, 136.7, 130.4, 129.6, 129.4, 127.3, 125.9, 124.6, 123.6, 122.5, 120.9, 120.3, 118.5, 112.2, 68.1, 42.0, 39.5, 39.2, 37.6, 29.7, 29.5, 26.21, 26.20, 25.7, 25.3, 22.8, 21.5, 16.0. HRMS (ESI-TOF) m/z: Calcd for C_35_H_45_N_4_O_3_ [M + H]^+^ 569.3486, Found: 569.3461.

*N-((3s,5s,7s)-adamantan-1-yl)-2-phenyl-2H-indazole-3-carboxamide (****4c****)*^1^

56.6 mg, 76%; White solid, m.p. 199‑200 °C; ^1^H NMR (600 MHz, CDCl_3_) δ 7.92 (d, *J* = 8.4 Hz, 1H), 7.79 (d, *J* = 9.0 Hz, 1H), 7.62 ‒ 7.61 (m, 2H), 7.56 ‒ 7.51 (m, 3H), 7.38 ‒ 7.36 (m, 1H), 7.25 – 7.23 (m, 1H), 5.48 (s, 1H), 2.08 (s, 3H), 1.99 (d, *J* = 2.4 Hz, 6H), 1.69 – 1.68 (m, 6H). ^13^C NMR (150 MHz, CDCl_3_) δ 158.7, 148.5, 140.2, 130.3, 129.6, 129.4, 127.2, 125.9, 124.4, 122.4, 120.4, 118.4, 53.1, 41.7, 36.4, 29.5. HRMS (ESI-TOF) m/z: Calcd for C_24_H_26_N_3_O [M + H]^+^ 372.2070, Found: 372.2058.

*N-(((1R,4aS,10aR)-7-isopropyl-1,4a-dimethyl-1,2,3,4,4a,9,10,10a-octahydrophenanthren-1-yl)methyl)-2-phenyl-2H-indazole-3-carboxamide (****4d****)*

40.0 mg, 40%; White solid, m.p. 104-105 °C; ^1^H NMR (600 MHz, CDCl_3_) δ 8.03 (d, *J* = 8.4 Hz, 1H), 7.78 (d, *J* = 9.0 Hz, 1H), 7.52 (d, *J* = 7.8 Hz, 2H), 7.39 – 7.36 (m, 1H), 7.31 (t, *J* = 7.8 Hz, 2H), 7.25 – 7.24 (m, 1H), 7.22 – 7.20 (m, 1H), 7.15 (d, *J* = 8.4 Hz, 1H), 7.01 (d, *J* = 8.4 Hz, 1H), 6.88 (s, 1H), 5.65 (t, *J* = 6.0 Hz, 1H), 3.30 (d, *J* = 6.6 Hz, 2H), 2.88 – 2.83 (m, 2H), 2.67 – 2.61 (m, 1H), 2.25 (d, *J* = 12.6 Hz, 1H), 1.79 – 1.59 (m, 4H), 1.29 – 1.24 (m, 8H), 1.18 (s, 3H), 1.09 – 1.02 (m, 2H), 0.91 (s, 3H). ^13^C NMR (150 MHz, CDCl_3_) δ 159.9, 148.6, 147.1, 145.9, 140.2, 134.7, 130.0, 129.7, 129.5, 127.3, 127.0, 126.2, 124.8, 124.3, 124.0, 123.2, 120.9, 118.3, 50.2, 45.2, 38.3, 37.5, 37.4, 36.2, 33.6, 30.1, 25.4, 24.23, 24.16, 19.0, 18.9, 18.6. HRMS (ESI-TOF) m/z: [M + H]^+^ Calcd for C_34_H_39_N_3_NaO^+^ 528.2985, Found: 528.2999.

*methyl (2-phenyl-2H-indazole-3-carbonyl)-L-leucinate (****4e****)*

46.0 mg, 63%; White solid, m.p. 129‑130 °C; ^1^H NMR (600 MHz, CDCl_3_) δ 8.00 (d, *J* = 8.4 Hz, 1H), 7.83 (d, *J* = 9.0 Hz, 1H), 7.63 – 7.60 (m, 2H), 7.56 – 7.52 (m, 3H), 7.42 – 7.39 (m, 1H), 7.32 – 7.30 (m, 1H), 6.22 (d, *J* = 8.4 Hz, 1H), 4.82 – 4.78 (m, 1H), 3.76 (s, 3H), 1.72 – 1.67 (m, 1H), 1.63 – 1.58 (m, 1H), 1.54 – 1.51 (m, 1H), 0.96 – 0.92 (m, 6H). ^13^C NMR (150 MHz, CDCl_3_) δ 173.2, 159.4, 148.6, 140.2, 129.7, 129.4, 128.6, 127.3, 126.0, 125.0, 122.8, 120.2, 118.6, 52.6, 51.1, 41.6, 25.1, 22.9, 22.0. HRMS (ESI-TOF) m/z: Calcd for C_21_H_24_N_3_O_3_ [M + H]^+^ 366.1812, Found: 366.1822.

*methyl (2-phenyl-2H-indazole-3-carbonyl)-L-phenylalanyl-L-leucinate (****4f****)*

75.0 mg, 73%; White solid, m.p. 190-191 °C; ^1^H NMR (600 MHz, CDCl_3_) δ 7.80 (d, *J* = 8.4 Hz, 1H), 7.66 (d, *J* = 8.4 Hz, 1H), 7.48 (s, 5H), 7.38 – 7.35 (m, 1H), 7.32 – 7.28 (m, 3H), 7.23 – 7.21 (m, 3H), 6.66 (d, *J* = 7.2 Hz, 1H), 6.14 (d, *J* = 7.8 Hz, 1H), 4.89 – 4.86 (m, 1H), 4.58 – 4.54 (m, 1H), 3.72 (s, 3H), 3.14 (d, *J* = 7.2 Hz, 2H), 1.62 – 1.57 (m, 2H), 1.48 – 1.44 (m, 1H), 0.89 – 0.86 (m, 6H). ^13^C NMR (100 MHz, CDCl_3_) δ 172.8, 170.3, 159.4, 148.5, 140.3, 136.2, 129.54, 129.49, 129.3, 129.0, 128.1, 127.4, 127.2, 125.9, 125.0, 122.4, 119.9, 118.6, 54.7, 52.5, 51.1, 41.6, 38.3, 24.9, 22.8, 22.0. HRMS (ESI-TOF) m/z: Calcd for C_30_H_33_N_4_O_4_ [M + H]^+^ 513.2496, Found: 513.2470.

*benzyl (2-phenyl-2H-indazole-3-carbonyl)glycyl-L-prolyl-L-phenylalaninate (****4g****)*

51.0 mg, 40%; Light yellow oil; ^1^H NMR (600 MHz, CDCl_3_) δ 8.04 (d, *J* = 9.0 Hz, 1H), 7.86 (d, *J* = 9.0 Hz, 1H), 7.61 (d, *J* = 7.2 Hz, 2H), 7.54 – 7.49 (m, 3H), 7.43 – 7.40 (m, 1H), 7.37 – 7.28 (m, 6H), 7.15 – 7.11 (m, 3H), 7.01 – 7.00 (m, 2H), 5.22 – 5.15 (m, 2H), 4.90 (q, *J* = 6.6 Hz, 1H), 4.55 (d, *J* = 8.4 Hz, 1H), 4.21 – 4.17 (m, 1H), 4.04 – 4.01 (m, 1H), 3.37 – 3.30 (m, 2H), 3.22 – 3.19 (m, 1H), 3.03 – 3.00 (m, 1H), 2.37 – 2.34 (m, 1H), 2.05 – 1.96 (m, 2H), 1.86 – 1.79 (m, 1H). ^13^C NMR (150 MHz, CDCl_3_) δ 171.3, 170.2, 167.7, 159.4, 148.6, 140.5, 136.0, 135.3, 129.6, 129.4, 129.3, 128.8, 128.71, 128.69, 128.4, 128.3, 127.2, 127.1, 126.1, 125.2, 122.4, 120.1, 118.8, 67.4, 60.1, 53.2, 46.3, 42.5, 37.8, 27.3, 24.8. HRMS (ESI-TOF) m/z: Calcd for C_37_H_36_N_5_O_5_ [M + H]^+^ 630.2711, Found: 630.2687.

*methyl (2-phenyl-2H-indazole-3-carbonyl)glycylphenylalanylleucinate (****4h****)*

60.0 mg, 53%; Light yellow oil; ^1^H NMR (400 MHz, CDCl_3_) δ 7.95 (d, *J* = 8.8 Hz, 1H), 7.83 (d, *J* = 8.8 Hz, 1H), 7.59 – 7.56 (m, 2H), 7.53 – 7.49 (m, 3H), 7.41 – 7.37 (m, 1H), 7.30 – 7.28 (m, 1H), 7.24 – 7.17 (m, 5H), 6.79 – 6.75 (m, 2H), 6.19 (d, *J* = 8.0 Hz, 1H), 4.69 (q, *J* = 7.2 Hz, 1H), 4.51 – 4.46 (m, 1H), 4.06 (d, *J* = 5.2 Hz, 2H), 3.66 (s, 3H), 3.10 – 3.00 (m, 2H), 1.53 – 1.39 (m, 3H), 0.86 – 0.83 (m, 6H). ^13^C NMR (100 MHz, CDCl_3_) δ 172.8, 170.3, 168.2, 160.0, 148.5, 140.3, 136.2, 129.6, 129.5, 129.3, 128.8, 128.1, 127.29, 127.26, 126.0, 125.1, 122.6, 120.1, 118.7, 54.7, 52.5, 51.1, 43.3, 41.5, 38.5, 24.9, 22.8, 22.1. HRMS (ESI-TOF) m/z: Calcd for C_32_H_36_N_5_O_5_ [M + H]^+^ 570.2711, Found: 570.2729.

## NMR copies of product

## References

1. V. S. Bhat, A. Lee, Direct C3 Carbamoylation of 2H-Indazoles[J], *Eur. J. Org. Chem*. **2021**, 23, 3382-3385.
